# Enhancing Care Transitions with an Explicit Live Discharge Protocol (LDP) from Hospice

**DOI:** 10.1093/geroni/igaf122.806

**Published:** 2025-12-31

**Authors:** Stephanie P Wladkowski, Susan Enguidanos, Tracy Schroepfer

**Affiliations:** Bowling Green State University, Bowling Green, Ohio, United States; University of Southern California, Los Angeles, California, United States; University of Wisconsin–Madison, Madison, Wisconsin, United States

## Abstract

Implementing a live discharge protocol (LDP) from hospice care is a critical component of ensuring safe and compassionate transitions for hospice patients (and their caregivers) who no longer meet eligibility criteria. A live discharge protocol (LDP) for hospice was recently developed with aims to (1) enhance patient and caregiver experience, (2) minimize disruptions of care, and (3) support hospice providers in navigating the complexities of care transitions. Key elements of the LDP include an inventory of clinical needs, assessment of psychosocial and financial concerns, needed community referrals, and post-discharge follow-up to prevent care gaps and assess caregiving well-being post-discharge. We tested the LDP in 4 US hospice agencies for live discharges experienced due to extended prognosis (n = 30). Following each discharge, in-depth, semi-structured interviews were conducted with hospice providers to elicit their perspectives on the usability of the LDP and suggestions for improving LDP implementation. A second set of interviews (n = 19) were conducted after the post-discharge phone call to explore the clinicians’ experiences with the LDP process. Thematic analysis was used. Providers noted the LDP was useful in ensuring a thorough collection of currently used equipment and supplies for improved discharge process. The need for the LDP referral section was specifically valuable for patients living at home. Finally, the follow-up phone call was found to be useful to support the hospice clinician in this care transition, and as a mechanism to remain connected to patients/caregivers for potential future re-enrollment onto hospice. Future research, policy and practice implications will be discussed.

